# Atrio-ventricular deformation and heart failure in Ebstein's Anomaly — A cardiovascular magnetic resonance study

**DOI:** 10.1016/j.ijcard.2017.11.097

**Published:** 2018-04-15

**Authors:** Michael Steinmetz, Marike Broder, Olga Hösch, Pablo Lamata, Shelby Kutty, Johannes T. Kowallick, Wieland Staab, Christian Oliver Ritter, Gerd Hasenfuß, Thomas Paul, Joachim Lotz, Andreas Schuster

**Affiliations:** a Department of Pediatric Cardiology and Intensive Care Medicine, University Medical Center, Georg-August-University Göttingen, Heart Center, Germany; b Inst. for Diag. and Interventional Radiology, University Medical Center, Georg-August-University Göttingen, Heart Center, Germany; c Department of Cardiology and Pneumology, University Medical Center, Georg-August-University Göttingen, Germany; d DZHK, German Center for Heart Research, partner site Göttingen, Germany; e University of Nebraska Medical Center/Children's Hospital and Medical Center, Omaha, NE, USA; f Department of Computer Science, University of Oxford, Oxford, United Kingdom,; g Division of Imaging Sciences and Biomedical Engineering, The Rayne Institute, St. Thomas' Hospital, King's College London, London, United Kingdom; h Department of Cardiology, Royal North Shore Hospital, The Kolling Institute, Northern Clinical School, University of Sydney, Sydney, Australia

**Keywords:** Ebstein's Anomaly, CMR, Feature tracking, Strain, R/L-Volume-Index, Heart failure

## Abstract

**Purpose:**

We aimed to quantify atrial and ventricular myocardial deformation in Ebstein's Anomaly (EA) in a case-control study with cardiovascular magnetic resonance (CMR) feature tracking and to correlate changes in cardiac performance with the severity of disease and clinical heart failure parameters.

**Materials and methods:**

Atrial and ventricular deformation was measured using CMR feature tracking in 30 EA and 20 healthy control subjects. Atrial performance was characterized using longitudinal strain and strain rate parameters for reservoir function, conduit function and booster pump function. Ventricular performance was characterized using RV and LV global longitudinal strain (εl) and LV circumferential and radial strain (εc and εr). Volumetric measurements for the ventricles including the Total Right/Left-Volume-Index (R/L-Volume-Index) and heart failure markers (BNP, NYHA class) were also quantified.

**Results:**

EA patients showed significantly impaired right atrial performance, which correlated with heart failure markers (NYHA, BNP, R/L-Volume-Index). LA function in EA patients was also impaired with atrial contractile function correlating with NYHA class. EA patients exhibited impaired RV myocardial deformation, also with a significant correlation with heart failure markers.

**Conclusion:**

CMR feature tracking can be used to quantify ventricular and atrial function in a complex cardiac malformation such as EA. EA is characterized by impaired quantitative right heart atrio-ventricular deformation, which is associated with heart failure severity. While LV function remains preserved, there is also significant impairment of LA function. These quantitative performance parameters may represent early markers of cardiac deterioration of potential value in the clinical management of EA.

## Highlights

• CMR feature tracking can be performed in complex congenital heart disease as Ebstein's Anomaly.
• Ebstein's Anomaly is associated with impaired right atrial and right ventricular strain markers.
• LA function as expressed by strain markers from CMR is also impaired.
• Atrio-ventricular deformation markers may be helpful in the management of Ebstein's Anomaly.

## 1 Introduction

Ebstein's Anomaly (EA) of the tricuspid valve (TV) accounts for approximately 0.5% of congenital heart disease (CHD) patients. Many EA patients suffer from progressive right heart failure and impaired physical exercise capacity at some stage in their life [1], [2]. The pathophysiology comprises a dysplastic and tethered TV displaced towards the right ventricular (RV) apex with subsequent “atrialization” of the RV (aRV), higher grade tricuspid valve regurgitation (TVR) and RA and RV volume overload [3]. Additionally, deterioration of LV function is frequently observed in EA [4], [5]. To date, the pathophysiological changes that ultimately cause progression to global heart failure in EA are unknown. Moreover there is a lack of biomarkers of clear clinical value that indicate cardiac deterioration at an early stage in EA.

The clinical relevance of impaired atrial function in heart failure development is increasingly recognized [6]. Right atrial (RA) function has been studied in some congenital heart disease (CHD) [7], [8] using echocardiography and CMR [9]. Both echocardiography and CMR-FT are equally able to discriminate left and right atrial phasic performance based on strain (ε) and strain rate (SR) imaging [10], [11], [12]. The reservoir phase, characterized by collection of pulmonary venous return during ventricular systole can be quantified by global peak longitudinal total strain (εs) and global peak positive SR (SRs). The conduit phase that describes the passage of blood to the ventricle during early ventricular diastole is indicated by global longitudinal passive strain (εe) and global peak early negative SE (SRe). The contractile phase or booster pump function describes the intensity of atrial contraction resulting in augmentation of ventricular filling during late ventricular diastole and can be quantified measuring global longitudinal active strain (εa) and global peak late-negative SR (SRa) during atrial contraction [12]. These parameters have been studied in non-symptomatic participants of the Multi-Ethnic-Study of Arteriosclerosis [13], [14] demonstrating prognostic value in heart failure prediction and in patients with atrial fibrillation showing a close link to stroke occurrence.

Alterations in atrial function – which reflect changes in myocardial deformation itself but are also dependent on RV function, loading conditions and tricuspid regurgitation – may possibly represent early markers for deterioration of overall cardiac function, but have not been defined in EA [11]. Also the interplay of atrial and ventricular performance of the right and left heart may play a role in EA disease progression. Thus, we sought to assess atrial and ventricular function in EA using a comprehensive CMR protocol and myocardial FT analysis. Moreover, we aimed to correlate changes in biatrial and biventricular performance with the severity of EA and clinical parameters of heart failure.

## 2 Methods

### 2.1 Study population

From a total of fifty-eight patients with EA in the database of the Department of Pediatric and Adult CHD, University Medical Center Göttingen, Germany, 31 patients with EA were prospectively recruited in this case-control study and gave written informed consent to participate. Exclusion criteria included age < 10 years, implantable electronic cardiac devices, associated complex cardiac malformations and claustrophobia. The 31 patients (21 male) were examined prospectively within one day between January 2013 and July 2013 and compared to 20 healthy matched controls (10 male). Six of the EA patients had undergone previous cardiac surgery (2 Glenn-Anastomosis, 2 TV reconstructions, one TV replacement and one patient with both, a TV reconstruction and Glenn-Anastomosis). Seven of eight patients with atrial septum defect (ASD) had undergone ASD closure (3 interventional, 4 surgical). Sinus rhythm was documented during the examination and no higher degree AV blocks were noted. The study protocol included medical history, New York Heart Association (NYHA) classification, clinical examination, brain natriuretic peptide (BNP) and CMR. The study protocol complied with the Declaration of Helsinki and was approved by the local clinical ethics committee.

### 2.2 CMR imaging

CMR was performed according to current guidelines [15]. All CMR measurements were performed at a 1.5 Tesla MRT-“Symphony“-scanner (Siemens Medical Systems, Erlangen, Germany). All patients were examined according to a standardized imaging protocol for EA and without sedation. A 16 channel phased-array-receiver coil was used for signal reception. Ventricular dimensions and function were assessed using stacks of multislice-multiphase steady state free precession cine images in ventricular short-axis as well as in axial orientations. Standard imaging parameters were as follows: repetition time = 44 ms, echo time = 1.3 ms, flip angle = 20°, slice thickness = 5 mm, spatial resolution 1.6 × 1.6 mm; parallel imaging acceleration factor 2; 12 s breath hold; with retrospective gating. Pulmonary artery flow was measured using phase contrast through-plane measurements in the main pulmonary artery just above the pulmonary valve and then used to calculate the TVR with the equation introduced by Fratz et al. $\left(\mathrm{TR}=\left(\frac{f\mathrm{RVSV}-\mathrm{PAante}}{f\mathrm{RVSV}-\mathrm{PAretro}}\right)\times 100\right)$ [16].

### 2.3 Feature tracking

Myocardial FT was performed using dedicated software (TomTec Imaging Systems, 2D CPA MR, Cardiac Performance Analysis, Version 1.1.2.36, Unterschleissheim, Germany). Results are based on averaging of three repeated measurements by the same observer to maximize reproducibility as previously demonstrated [17].

### 2.4 Atrial feature tracking analysis

The 4-chamber view showing all 4 cardiac chambers was used to calculate LA and RA strain and strain rate [18]. As the anatomy can vary significantly in EA patients this will be referred to as the horizontal long axis view in the course of this manuscript. Atrial endocardial borders were manually drawn using a point-and-click approach at the end of ventricular diastole representing the time of the lowest atrial filling. Atrial borders of the anatomical RA were tracked at the site of the true tricuspid annulus from the lateral wall to the septal RA wall. We thereby excluded the atrialized portion of the RA, which is marked by the displaced tricuspid valve and could not be tracked. After the automated tracking algorithm was applied, tracking performance was visually controlled and, in case of insufficient border tracking, manually corrected and the algorithm reapplied. In case of persisting insufficient tracking quality, the corresponding segment was excluded from the analysis.

**Fig. 1:**
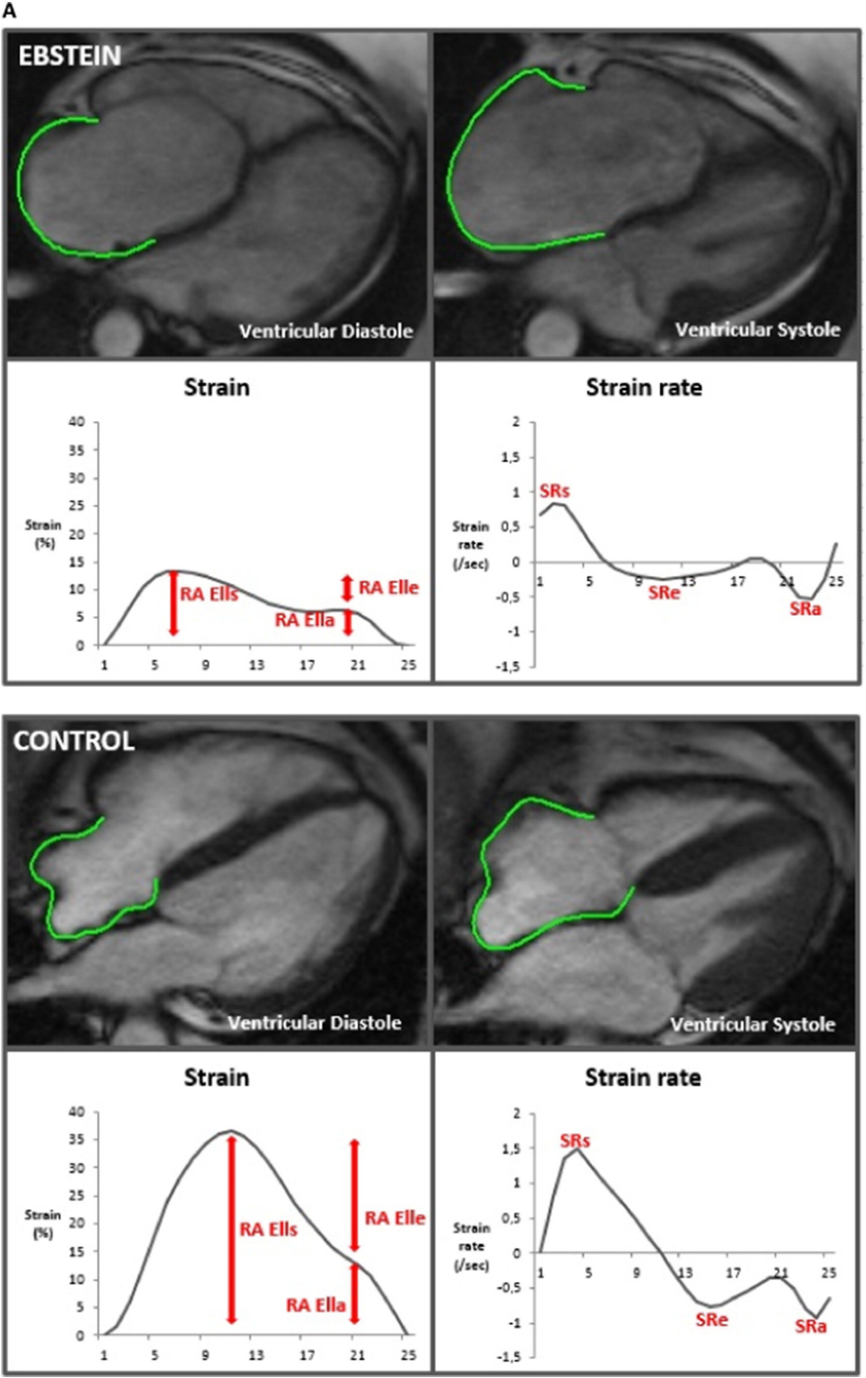

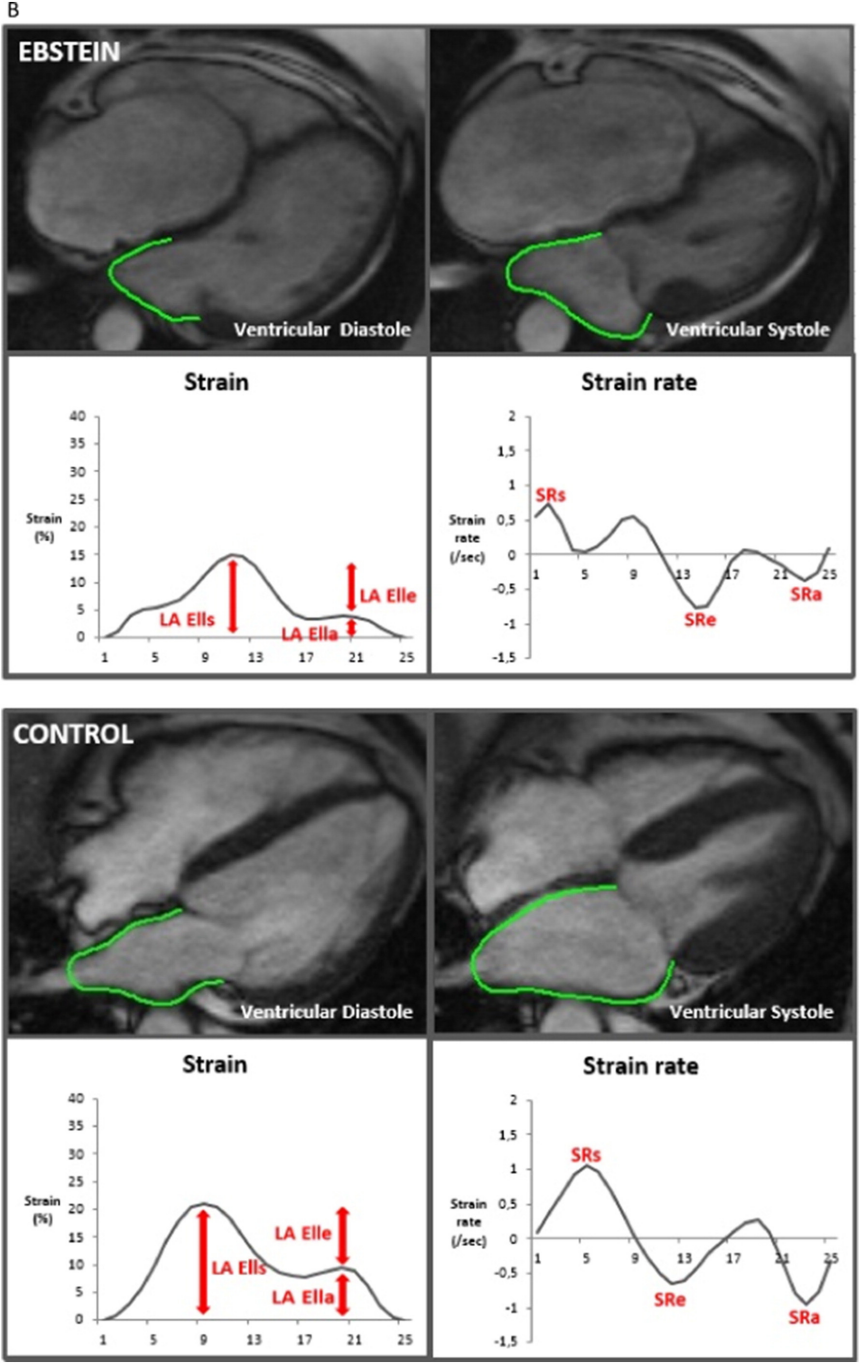
A: Right atrial feature tracking in a patient with Ebstein's Anomaly and a healthy control. B: Left atrial feature tracking in a patient with Ebstein's Anomaly and a healthy control. RA εl: right atrial longitudinal strain; LA εl: left atrial longitudinal strain; SR: strain rate.

The three main components of atrial physiology were quantified as follows (Figs. 1A and B): 1) atrial reservoir function representing the collection of venous return during ventricular systole using total strain (RA εs/LA εs) and peak positive strain rate (SRs), 2) atrial conduit function representing the passage of blood to the ventricles during early ventricular diastole using passive strain (RA εe/LA εe) and peak early negative strain rate (SRe) and 3) atrial contractile booster pump function representing the augmentation of ventricular filling during late ventricular diastole using active strain (RA εa/LA εa) and peak late negative strain rate (SRa) [6].

### 2.5 Ventricular analysis

Ventricular strain was calculated from horizontal long axis and short-axis slices as previously described [19]. The horizontal long axis was used to quantify endocardial RV and LV global longitudinal strain (RV εl/LV εl). In order to calculate global circumferential and radial LV short-axis strain (LV εc and LV εr), endo- and epicardial contours, were drawn on basal, mid-ventricular and apical short-axis slices. Global LV εc was calculated as average from subendocardial and subepicardial LV εc.

Volumetric analysis was performed with commercially available software (QMass, Medis, The Netherlands). For EA patients the functional right ventricle (fRV) was used for RV measurements and contours were drawn to the insertion of the displaced TV leaflets. For ventricular volumetric analysis, endocardial contours for RV and LV were drawn in all short axis slices at end systole and end diastole. Short axis orientation was employed deliberately since previous work has demonstrated similar results for short axis and transverse quantification of ventricular function [20], as well as to ensure comparability between EA patients and healthy volunteers. EF was calculated from end diastolic volume indexed to BSA (EDVi) and end systolic volume indexed to BSA (ESVi).

The Total Right/Left-Volume-Index (R/L-Volume-Index) as a clinical severity parameter of EA was calculated from end diastolic volume measurements $\left(R/L‐\text{Volume}‐\text{Index}=\frac{\mathrm{RA}+\mathrm{aRV}+f\mathrm{RV}}{\mathrm{LA}+\mathrm{LV}}\right)$ [20].

### 2.6 Statistical analysis

Statistical analysis was performed using Microsoft Excel (Microsoft Corporation, Redmond, Washington, USA), Statistica (Stat Soft, North Melbourne, Australia) and IBM SPSS Statistics version 22 (IBM Corporation, Armonk, New York, USA). Data are expressed as mean (± standard deviation). After assessment for normality using the Kolmogorov-Smirnov test, the data were compared using a *t*-test and the Mann-Whitney-*U* test for parametric and non-parametric data, respectively. Spearman's correlation coefficients were calculated for relations between functional indexes and heart failure parameters and for relations between the functional indexes of atria and ventricles. A sub-group analysis was performed after exclusion of EA patients with previous corrective surgery (24 patients left).

Measurements were performed by two independent skilled observers. The first observer (M.B) analysed all data based on average curves of three analyses repetitions and repeated the analyses of the functional atrial parameters in 10 randomly selected subjects after a period of 4 weeks for the intra-observer variability. The second observer (J.T.K.) performed the analysis in the same 10 subjects for the analysis of the inter-observer variability. Reproducibility was assessed using the coefficient of variation [21] and the method described by Bland and Altman [22]. Due to multiple comparisons, *p*-values for Spearman's correlation were reported for descriptive purposes. For all other comparisons, *p*-values < 0.05 were considered statistically significant.

## 3 Results

CMR-FT was successfully performed in 30 patients with EA and all controls. One patient with EA was excluded due to insufficient image quality from motion artifacts. The mean age of the EA patients was 26.3 years (controls 23.7 years, *p* = 0.238).

Overall, 85.3% of the atrial segments were included in the EA group and 94.6% in the control group. Exclusion of atrial segments was predominantly associated with poor tracking quality due to insertion of pulmonary veins in the LA and poor imaging quality associated with breathing motion.

### 3.1 Right atrial function

RA reservoir and booster pump function were significantly reduced in the EA group as compared to controls (Table 1). Conduit function was significantly lower in EA based on SRe, whilst there was a trend towards reduction in εe derived conduit function (Table 1).

**Table 1:** Comparison of functional parameters of EA patients and healthy controls.

	EA patients	Controls	*P*-value	EA patients	Controls	*P*-value
	*Right atrium*			*Left atrium*		
*Reservoir function*						
Strain (εs (%))	20.50 ± 9.91	28.79 ± 10.86	**0.009**	17.81 ± 9.45	21.06 ± 5.79	**0.037**
Strain rate (SRs (/s))	0.96 ± 0.35	1.20 ± 0.42	**0.037**	0.74 ± 0.31	0.89 ± 0.27	0.094

*Conduit function*						
Strain (εe (%))	14.31 ± 7.93	18.29 ± 8.85	0.094	13.14 ± 7.95	15.28 ± 5.30	**0.040**
Strain rate (SRe (/s))	− 0.65 ± 0.29	− 0.90 ± 0.42	**0.021**	− 0.76 ± 0.38	− 1.08 ± 0.44	**0.007**

*Booster pump function*						
Strain (εa (%))	6.19 ± 4.88	10.49 ± 5.27	**0.006**	4.67 ± 3.38	5.78 ± 3.76	0.300
Strain rate (SRa (/s))	− 0.59 ± 0.40	− 0.90 ± 0.48	**0.020**	− 0.41 ± 0.32	− 0.61 ± 0.35	**0.047**

	*Right ventricle*			*Left ventricle*		
EDVi (ml/m^2^)	117.54 ± 58.10	76.13 ± 16.97	**< 0.001**	72.35 ± 10.42	76.60 ± 10.19	0.172
ESVi (ml/m^2^)	66.85 ± 40.00	36.22 ± 9.09	**< 0.001**	29.64 ± 6.82	28.81 ± 8.14	0.708
EF total (%)	44.77 ± 8.33	52.99 ± 5.23	**< 0.001**	59.07 ± 7.50	62.75 ± 7.67	0.281
Longitudinal strain (εl (%))	− 13.48 ± 6.26	− 19.66 ± 3.60	**< 0.001**	− 15.67 ± 4.96	− 18.37 ± 4.78	0.067
Circumferential strain (εc (%))				− 17.61 ± 4.63	− 17.81 ± 3.13	0.870
Radial strain (εr (%))				25.65 ± 9.83	28.22 ± 9.75	0.378

*P*-value from *t*-test or Mann-Whitney-*U* test, bold *p*-values indicate statistical significance.

EA: Ebstein's Anomaly; AVI: atrial volume, indexed; EF: ejection fraction; indexed; ε: strain; SR: strain rate; EDVi: end-diastolic volume, ESVi: end-systolic volume, indexed; εc: circumferential strain; εr: radial strain.

#### 3.1.1 Correlation with heart failure markers

RA function exhibited correlations with heart failure parameters in EA patients (see Table 2): 1) atrial reservoir function correlated significantly with BNP level (RA εs and SRs) and NYHA class (*p* < 0.05 for RA εs; and a strong trend for RA SRs, *p* = 0.067); 2) atrial conduit function (RA εe and SRe) correlated with the R/L-Volume Index; and 3) atrial booster pump function (RA εa and SRa) correlated with NYHA class and BNP (see Table 2).

**Table 2:** Correlation of right atrial and ventricular volumes and functional indexes from MR with heart failure parameters of EA patients.

	R/L-Volume-Index	NYHA	BNP
*Right atrium*			
Reservoir function			
Strain (εs)	**− 0.449 (0.019)**	**− 0.446 (0.015)**	**− 0.637 (< 0.001)**
Strain rate (SRs)	n.s.	− 0.345 (0.067)	**− 0.487 (0.009)**
Conduit function			
Strain (εe)	**− 0.416 (0.031)**	n.s.	**− 0.516 (0.005)**
Strain rate (SRe)	**− 0.400 (0.039)**	n.s.	n.s.
Booster pump function			
Strain (εa)	n.s.	**− 0.560 (0.002)**	**− 0.420 (0.026)**
Strain rate (SRa)	n.s.	**− 0.476 (0.009)**	**− 0.409 (0.031)**

*Right ventricle*			
EDVi	**0.844 (< 0.001)**	n.s.	n.s.
ESVi	**0.918 (< 0.001)**	n.s.	n.s.
EF	**− 0.670 (< 0.001)**	n.s.	n.s.
Strain (εl)	**− 0.644 (< 0.001)**	**− 0.466 (0.012)**	**− 0.395 (0.042)**

Spearman's correlation coefficient (*p*-value), n.s.: non-significant.

RAVI: right atrial volume, indexed; EF: ejection fraction; ε: strain; SR: strain rate; EDVi: end-diastolic volume, ESVi: end-systolic volume, indexed; R/L-Volume-Index: Total Right/Left-Volume-Index; NYHA: New York Heart Association classification; BNP: brain natriuretic peptide.

There was no significant correlation between RA deformation parameters and the degree of TVR.

### 3.2 Right ventricular function

RV volume was higher, and RVEF and RV longitudinal strain (RV εl) were significantly lower in EA patients compared with healthy controls (see Table 1).

#### 3.2.1 Correlation with heart failure markers

RV volume and RVEF correlated with the R/L-Volume Index. RV εl correlated with all heart failure parameters (R/L-Volume Index, NYHA class, BNP) (see Table 2).

#### 3.2.2 Atrio-ventricular coupling

Atrio-ventricular coupling analysis revealed correlations of RV εl with atrial reservoir and conduit function (see Table 3).

**Table 3:** Correlation of right atrial strain parameters with right ventricular volumes and functional indexes from MR of EA patients.

	EDVi	ESVi	RV EF	RV Ell
*Right atrium*				
Reservoir function				
Strain (εs)	n.s.	n.s.	n.s.	**0.660 (< 0.001)**
Strain rate (SRs)	n.s.	n.s.	n.s.	**0.460 (0.014)**
Conduit function				
Strain (εe)	n.s.	n.s.	n.s.	**0.518 (0.005)**
Strain rate (SRe)	n.s.	n.s.	n.s.	**0.474 (0.011)**
Booster pump function				
Strain (εa)	n.s.	n.s.	n.s.	n.s.
Strain rate (SRa)	n.s.	n.s.	n.s.	n.s.

Spearman's correlation coefficient (*p*-value), n.s.: non-significant.

ε: strain; SR: strain rate; EDVi: end-diastolic volume, ESVi: end-systolic volume, indexed.

### 3.3 Left atrial function

EA patients had decreased LA reservoir (εs) and conduit function (εe, SRe) as well as reduced booster pump function (SRa) (see Table 1).

#### 3.3.1 Correlation with heart failure markers

LA booster pump function showed a correlation with NYHA class based on strain and SR, respectively (NYHA–LA εa *r* = − 0.455, *p* = 0.017 and SRa *r* = 0.455, p = 0.017).

### 3.4 Left ventricular function

There was a trend towards lower LV longitudinal strain (LV εl) in EA patients compared to controls (*p* = 0.067), while LV volumetric parameters did not differ between groups.

#### 3.4.1 Correlation with heart failure markers

LV volumetric data correlated with NYHA class and the R/L-Volume-Index (LVEF total–NYHA *r* = − 0.411, *p* = 0.030 and EDVi–R/L Index *r* = − 0.491, *p* = 0.009).

### 3.5 Impact of cardiac surgery

The correlation of heart failure stages with both reservoir function (NYHA, BNP) and booster pump function (NYHA) remained, irrespective of the exclusion of the patients who underwent corrective surgery (data not shown). Conversely, the association of RA booster pump function and BNP could not be detected when looking at the EA subgroup without previous cardiac surgery (*p* > 0.05).

Furthermore, cardiac surgery did not impact on the association of the severity of EA (R/L-Volume-Index) and RA conduit function.

### 3.6 Inter- and intraobserver variability

Atrial strain and SR were reproducible on an inter- and intraobserver level with low values for mean difference and coefficient of variation (range 10.5 to 32.8 depending on individual parameter, supplementary material supplied upon request).

## 4 Discussion

Our study describes a well-characterized, functional phenotype based on atrial and ventricular mechanical function in EA. We demonstrate that CMR-FT is a suitable technique for quantification of cardiac atrial and ventricular deformation in a complex congenital heart malformation such as EA. We can show for the first time, that in EA CMR-FT derived RA and RV function, but to some extent also LA function, was impaired and correlated with heart failure markers. Correlation analysis of RA function with heart failure markers revealed the following: 1) patients with more severe heart failure stages (expressed by BNP and NYHA class) showed larger reduction of RA reservoir function and RA booster pump function, and 2) patients with more severe EA (as expressed by the R/L-Volume-Index) had lower values for RA conduit function.

It is important to note, that these findings were independent of previous cardiac surgery and are thus applicable to a heterogeneous EA patient collective. Moreover, it is important to understand that RA function is dependent on loading conditions and RV and tricuspid valve function at the time of the exam. Changes in RA function are not necessarily reflective of overall intrinsic atrial function alone.

Besides functional impairment of the RA, LA conduit function was also affected to a lesser extent. Interestingly, whilst LA reservoir and conduit function were lower as compared to control subjects only LA booster pump function was related to heart failure severity as defined by NYHA. This is paralleled by association of reduced RA booster pump function with NYHA class in our cohort. Similar to findings in non-congenital left heart disease [23], this highlights the potential importance of the active atrial contractile function in heart failure in EA.

### 4.1 Right heart function

Standard RV functional parameters derived from CMR including EF and volumes do not correlate well with exercise capacity and heart failure markers in EA patients [24]. A lack of correlation between RV volumes and EF with NYHA and BNP was observed in the present study, as well, but we identified a correlation of RV strain and several RA metrics (Table 2).

Quantification of RA function in CHD is receiving increasing interest [25] and impaired RA function has been reported in congenital right heart disease Tetralogy of Fallot (TOF) [26] or ASD [27]. Reduced RA reservoir function and elevated conduit function occur in TOF with higher grade pulmonary regurgitation and large RV EDVi [26]. In our cohort of EA, RA reservoir and booster pump function were impaired and associated with higher NYHA class and increased BNP. This is in line with observations in TOF patients [9].

The mechanism behind impaired RA deformation might be a higher degree of RA preload (determined by a) central venous return and b) TR induced volume load) and an unfavourable pre-dilation of atrial myofibres (Frank-Starling mechanism). Changes in atrial strain and strain rate, as observed in our study, are reflective of contractility and distensibility of the myofibrils, i.e. possibly alterations in the optimal Frank-Starling curve. A study of Ren et al. links changes in LA function in patients with mitral regurgitation to the Frank Starling Mechanism and atrial pre-load conditions [28]. This supports our hypothesis that loading conditions and the Frank Starling mechanism play a role in altered atrial function in EA. Since the atrialized RV contracts in synchrony with the RV and thus against a closed TV, the degree of dyssynchrony of the aRV and anatomical RA may be another contributor to changes in the atrial contraction. This remains speculative, since aRV tracking is not feasible with our technique.

RV function in EA patients was also compromised, as expressed by impaired RV εl. Changes in RV εl depict changes in RV myocardial contraction and are – comparable to RA function – dependent on ventricular loading conditions. However, RV εl correlation with heart failure markers suggest that these changes in deformation may be clinically relevant.

Unifying the above observations, we hypothesized that cardiac dysfunction in EA is linked to decreased RA deformation – possibly based on a shift away from the optimal Frank-Starling curve – coupled with impaired RV longitudinal function (i.e. reduced RV εl in the EA group). Spearman analysis of RA–RV interaction in our cohort confirmed positive correlation of RV strain with RA reservoir and conduit function and supports this hypothesis.

### 4.2 Left heart function

Our results show that LA function is also affected, albeit to a lesser extent compared to the RA, while LV systolic function was normal. Even though systolic LV function may be preserved in our collective, LA reservoir and conduit function are reduced and may reflect LV diastolic dysfunction. This hypothesis is in line with Inai's description of diastolic dysfunction in EA with preserved LV EF [29].

### 4.3 Limitations

This was a single center cross sectional study. Our cohort only comprises a small number of patients with higher degree heart failure (NYHA III or IV), which is due to the low incidence of EA.

To discern the role of the RA conduit function further, CMR-FT analysis of both the RA and aRV would be necessary. However, since CMR-FT relies on tracking myocardial borders and the aRV lies within the fRV only separated from it by the malformed TV, there are no myocardial borders that can be tracked by CMR-FT. This makes CMR-FT analysis of the aRV technically impossible.

Finally, age-wise maturation of atrial function have been reported in the echocardiography literature [30]. Due to the rarity of the disease and the small patient numbers available, effects of age on atrial function have not been accounted for in the present study.

## 5 Conclusion

Right atrial and ventricular myocardial strain and strain rate are reduced in EA and can be reliably quantified using CMR-FT. CMR derived longitudinal right atrial and ventricular strain appear to be markers of heart failure in EA, since a close correlation of these parameters with NYHA class, BNP and R/L-Volume-Index was observed. Alterations in LA function were also noted. Incorporating atrial and ventricular deformation indices into CMR assessment may help to improve the management and decision making for intervention or operation in EA patients.

**Abbreviations**
aRV: atrialized right ventricle
ASD: atrial septum defect
BNP: brain natriuretic peptide
BSA: body surface area
CHD: congenital heart disease
CMR: cardiovascular magnetic resonance imaging
ε: strain
εc: circumferential strain
εl: longitudinal strain
εa: atrial active longitudinal strain
εe: atrial passive longitudinal strain
εs: atrial total longitudinal strain
εr: radial strain
EA: Ebstein's Anomaly
EDVi: end diastolic volume indexed to body surface area
EF: ejection fraction
ESVi: end systolic volume indexed to body surface area
fRV: functional right ventricle
FT: feature tracking
LA: left atrium
LV: left ventricle
NYHA: New York Heart Association
RA: right atrium
R/L-Volume-Index: Total-Right/Left-Volume-Index
RV: right ventricle
SR: strain rate
SRa: atrial peak late negative strain rate
SRe: atrial peak early negative strain rate
SRs: atrial peak positive strain rate
TOF: Tetralogy of Fallot
TVR: tricuspid valve regurgitation
TV: tricuspid valve

## Conflict of interest

The authors report no relationships that could be construed as a conflict of interest.

## Disclosures

None.

## Appendix A Supplementary data

Supplementary figures. Image 1

Supplementary video 1. Image 2
