# Supplementary material for: Atrio-ventricular deformation and heart failure in Ebstein's Anomaly — A cardiovascular magnetic resonance study
Source: Int J Cardiol. 2018 Apr 15;257:54–61. doi: 10.1016/j.ijcard.2017.11.097 (PMC5856281; doi:10.1016/j.ijcard.2017.11.097)
Supplement: Supplementary file 1 — Supplementary figures. [file mmc1.docx]

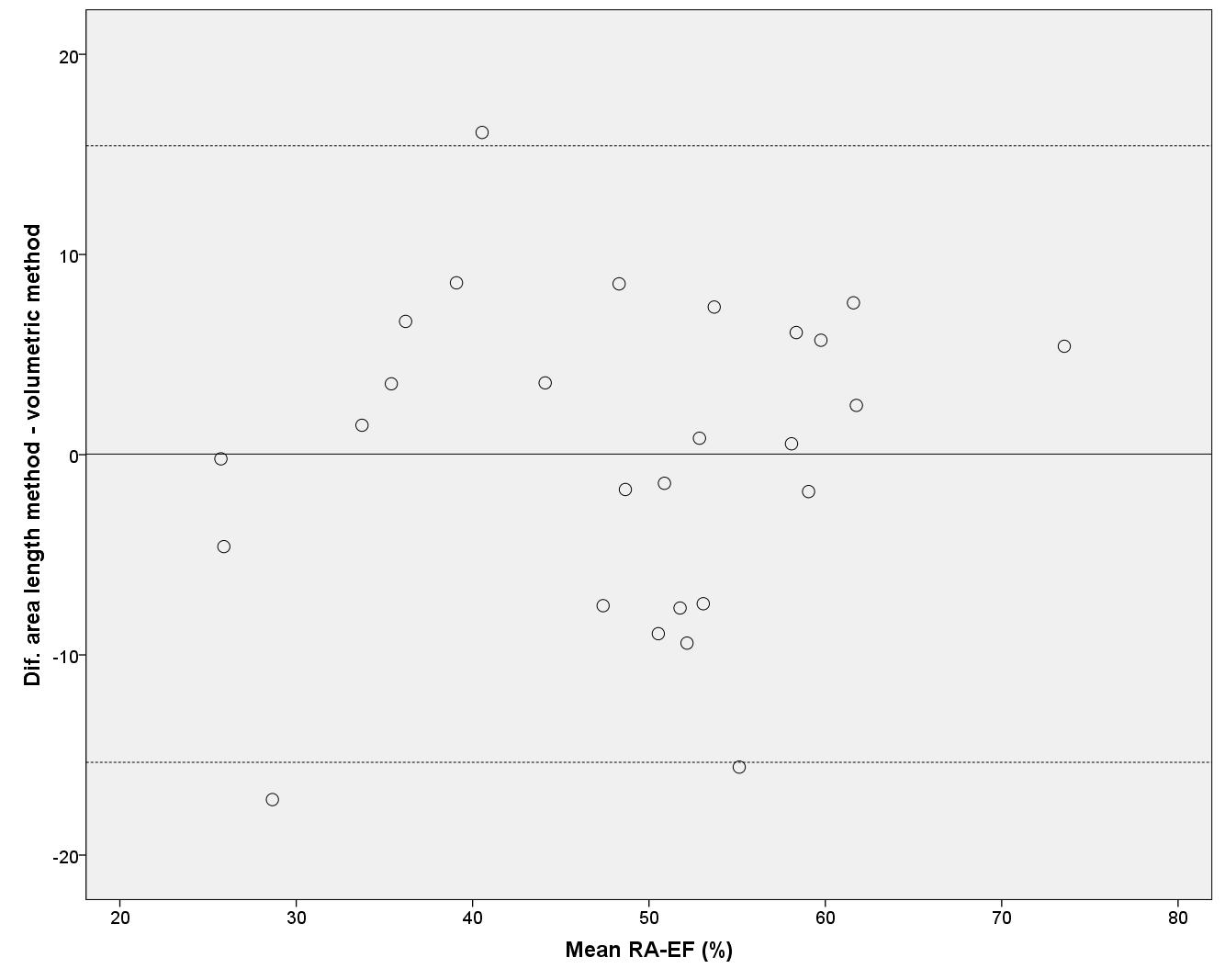


Figure 1 suppl. : Bland Altman plot of RA EF with limits of agreement (95% confidence intervals= dotted lines) and bias (black line) comparing volumetric and area length method.


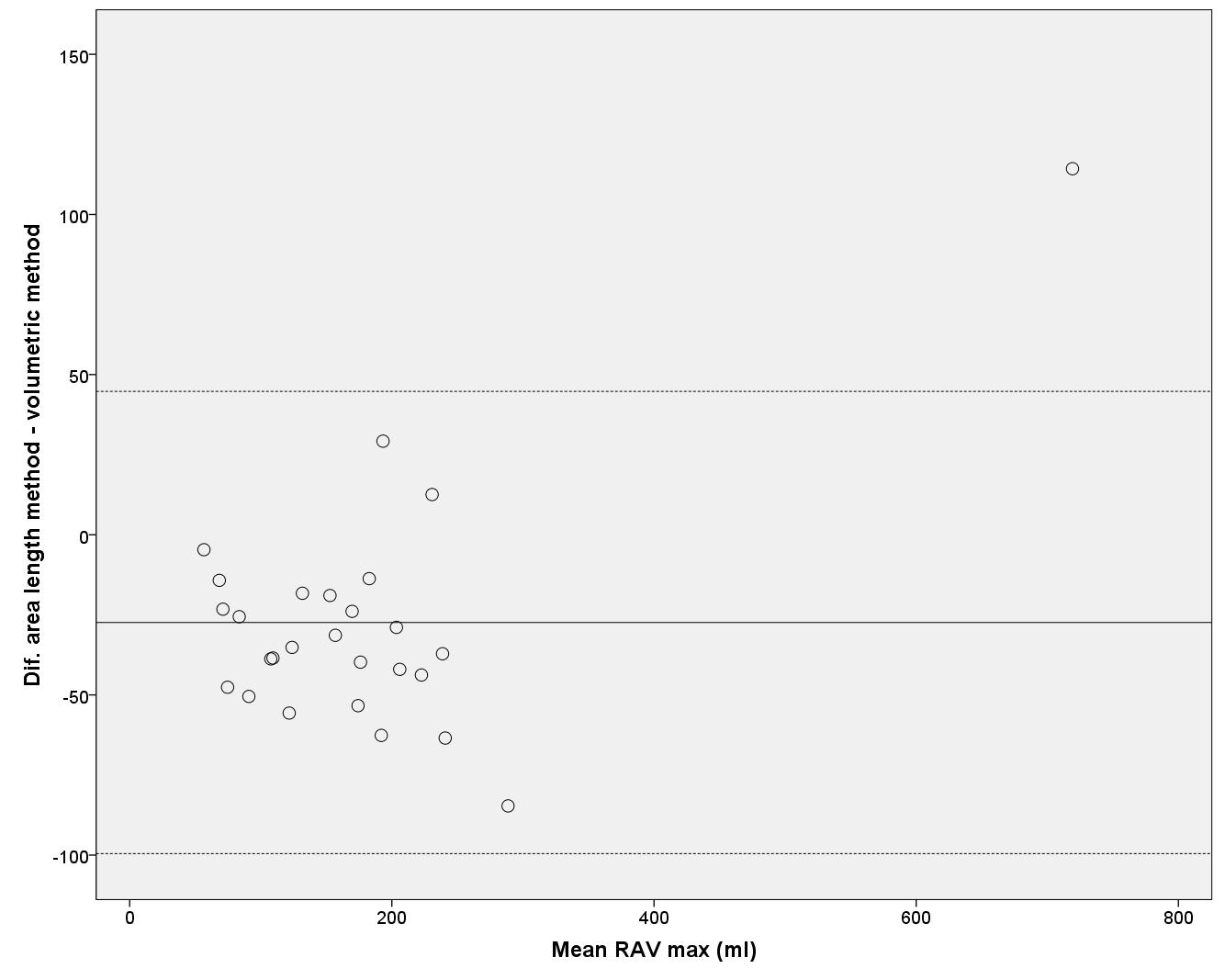


Figure 2 suppl. : Bland Altman plot of RA max. volume with limits of agreement (95% confidence intervals=dotted lines) and bias (black line)
